# Wavelet Entropy and Directed Acyclic Graph Support Vector Machine for Detection of Patients with Unilateral Hearing Loss in MRI Scanning

**DOI:** 10.3389/fncom.2016.00106

**Published:** 2016-10-19

**Authors:** Shuihua Wang, Ming Yang, Sidan Du, Jiquan Yang, Bin Liu, Juan M. Gorriz, Javier Ramírez, Ti-Fei Yuan, Yudong Zhang

**Affiliations:** ^1^School of Electronic Science and Engineering, Nanjing UniversityNanjing, China; ^2^School of Computer Science and Technology, Nanjing Normal UniversityNanjing, China; ^3^Hunan Provincial Key Laboratory of Network Investigational Technology, Hunan Police AcademyChangsha, China; ^4^Department of Radiology, Nanjing Children's Hospital, Nanjing Medical UniversityNanjing, China; ^5^Key Laboratory of Intelligent Computing and Information Processing in Fujian Provincial University, Quanzhou Normal UniversityQuanzhou, China; ^6^Jiangsu Key Laboratory of 3D Printing Equipment and ManufacturingNanjing, China; ^7^Department of Radiology, Zhong-Da Hospital of Southeast UniversityNanjing, China; ^8^Department of Signal Theory, Networking and Communications, University of GranadaGranada, Spain; ^9^State Key Lab of CAD & CG, Zhejiang UniversityHangzhou, China; ^10^Key Laboratory of Statistical Information Technology and Data Mining, State Statistics BureauChengdu, China

**Keywords:** unilateral hearing loss, sensorineural hearing loss, wavelet entropy, support vector machine, directed acyclic graph, confusion matrix, computer aided diagnosis

## Abstract

**Highlights**

1. We develop computer-aided diagnosis system for unilateral hearing loss detection in structural magnetic resonance imaging.
2. Wavelet entropy is introduced to extract image global features from brain images. Directed acyclic graph is employed to endow support vector machine an ability to handle multi-class problems.
3. The developed computer-aided diagnosis system achieves an overall accuracy of 95.1% for this three-class problem of differentiating left-sided and right-sided hearing loss from healthy controls.

**Aim:** Sensorineural hearing loss (SNHL) is correlated to many neurodegenerative disease. Now more and more computer vision based methods are using to detect it in an automatic way.

**Materials:** We have in total 49 subjects, scanned by 3.0T MRI (Siemens Medical Solutions, Erlangen, Germany). The subjects contain 14 patients with right-sided hearing loss (RHL), 15 patients with left-sided hearing loss (LHL), and 20 healthy controls (HC).

**Method:** We treat this as a three-class classification problem: RHL, LHL, and HC. Wavelet entropy (WE) was selected from the magnetic resonance images of each subjects, and then submitted to a directed acyclic graph support vector machine (DAG-SVM).

**Results:** The 10 repetition results of 10-fold cross validation shows 3-level decomposition will yield an overall accuracy of 95.10% for this three-class classification problem, higher than feedforward neural network, decision tree, and naive Bayesian classifier.

**Conclusions:** This computer-aided diagnosis system is promising. We hope this study can attract more computer vision method for detecting hearing loss.

## Introduction

Sensorineural hearing loss (SNHL) belongs to a type of hearing loss. The roots are located in either inner ear, or vestibulocochlear nerve, or central auditory processing center (Koylu et al., 2016). Among reported hearing loss, 90% are SNHLs. Its distinctive feature is that the loss usually falls in high-frequency region or a notch at some frequency (Lin et al., 2016). Only 2% of SNHLs have bilateral hearing impairments and most patients are unilateral hearing loss (UHL, Eftekharian and Amizadeh, 2016).

Except hearing loss (mild, moderate, severe, profound, or total), SNHL patients suffer from deficiency of diseases, especially brain functions. Take as examples, SNHL is shown to be correlated with lower intelligence (Martínez-Cruz et al., 2009), directional brain network, Meniere's Disease (Teranishi et al., 2012), neonatal hyperbilirubinemia (Khalid et al., 2015), motor proficiency (Martin et al., 2012), neurodevelopmental disorder (Chilosi et al., 2010), speech and language delay (Prosser et al., 2015), etc.

In the past, scholars have used fMRI and DTI to research the SNHL problems. Profant et al. (2014) used MR morphometry and DTI to study SNHL. Vaden et al. (2016) used fMRI to prove the trial-level word recognition benefit from cingulo-opercular activity was equivalent for both hearing loss groups. Li Z. et al. (2015) studied functional connectivity using rest-state fMRI. In all, either fMRI or DTI costs lengthy time for scan, hence, in this study, we aim to develop a computer-aided diagnosis (CAD) tool for automatically detecting left-sided hearing loss (LHL) and right-sided hearing loss (RHL) from healthy controls (HC) based on structural MRI (SMRI).

The tool can work since SNHLs have difference with healthy subjects in brain structures. Those alterations can be clearly found in advanced neuroimaging modalities, such as magnetic resonance imaging (MRI). Yang et al. (2014) proved UHL patients showed decreased gray matter volume in bilateral posterior cingulate gyrus and precuneus, left superior/middle/inferior temporal gyrus, and right parahippocampal gyrus and lingual gyrus. Hribar et al. (2014) proved manual volumetry revealed preserved GM volume of the bilateral HG and significantly decreased WM volume of the left HG in the deaf. Shiell et al. (2016) investigated the cortical thickness of cats, and found the right hemisphere planum temporale supports enhanced visual motion detection ability in deaf people.

Finally, CAD tools are not expected to replace otologists, but to assist them to make more accuracy diagnosis (Yuan, 2015; Amir and Lehmann, 2016; Choi et al., 2016). Computer vision, machine learning (Yang, 2016), and image processing (Zhang et al., 2016) techniques will be used to help us develop this CAD tool. To our best knowledge, this is the first study to develop CAD tool for SNHL detection based on SMRI. The rest of this paper is organized as follows. Section Materials provides the materials. Section Methodology offers the methodology. Section Results and Discussions gives the results and discussions. Finally, Section Conclusions concludes the paper.

## Materials

Our study consisted of 49 subjects: 20 HCs, 15 LHLs, and 14 RHLs. The inclusion criterion was moderate-to-severe sudden sensorineural UHL. The exclusion criteria for all participants were known neurological or psychiatric diseases, brain lesions such as tumors or strokes, taking psychotropic medications, and contraindications to MR imaging. This study was approved by the Ethics Committee of Southeast University, and a signed informed consent form was obtained from every subject prior to entering this study.

**Figure 1:**
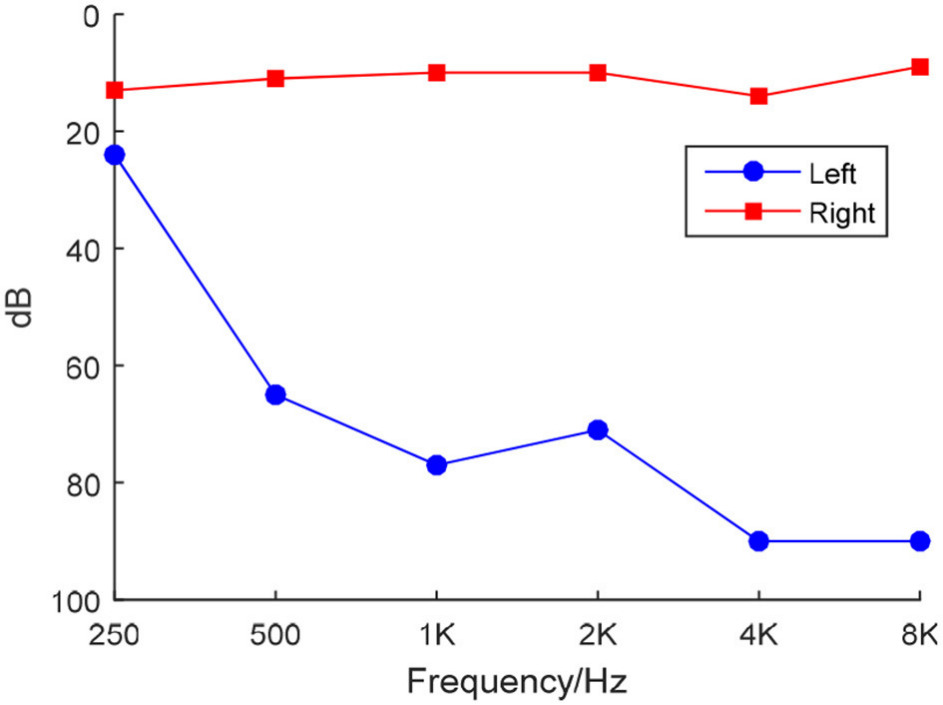
**Frequency-dependent hearing level of a LHL subject**.

We used a clinical audiometer to perform pure tone audiometry with six different octave frequencies (0.25, 0.5, 1, 2, 4, and 8 kHz), in order to measure the pure tone average (PTA) and reflect hearing performance (Yang et al., 2016). All patients were diagnosed with UHL with hearing deficit in either unilateral ear (PTA ≥ 40 dB) and normal hearing in both ears (PTA ≤ 25 dB). The patients included were all right-handed and 41 to 60 years old. For each patient, the hearing loss was sudden and persistent. None used a hearing aid on the impaired ear. Table 1 shows that the control group was well matched to the patient group in terms of age, sex, and education level. The audiogram of the affected ear of each patient is shown in Figure 1.

**Table 1:** **Demographic data of all subjects**.

	**LHL**	**RHL**	**Control**	***F/x^2^/t***	***P***
Age (year)	51.7 ± 9.6	53.9 ± 7.6	53.6 ± 5.4	0.305	0.739
Gender (m/f)	8/7	6/8	8/12		
Education level (year)	12.5 ± 1.7	12.1 ± 2.4	11.5 ± 3.2	0.487	0.618
Disease duration (year)	17.6 ± 17.3	14.2 ± 14.9	–	0.517	0.610
PTA of left ear (dB)	78.1 ± 17.9	21.8 ± 3.2	22.2 ± 2.1	156.427	0.00
PTA of right ear (dB)	20.4 ± 4.2	80.9 ± 17.4	21.3 ± 2.2	167.796	0.00

Data are mean ±SD, LHL, left-sided hearing loss; RHL, right-sided hearing loss; PTA, pure tone average; m, male; f, female; F/x^2^/t means the score calculated by F-test or Pearson's chi-squared test or Student's t-test.

Scanning was implemented by a Siemens Verio Tim 3.0T MR scanner (Siemens Medical Solutions, Erlangen, Germany). All subjects lie as still as possible with eyes closed and not to fall asleep. In total 176 sagittal slices covering the whole brain were acquired, using an MP-RAGE sequence. The imaging parameters were: TE = 2.48 ms, TR = 1900 ms, TI = 900 ms, FA = 9°, FOV = 256 × 256 mm, matrix = 256 × 256, slice thickness = 1 mm.

**Figure 2:**
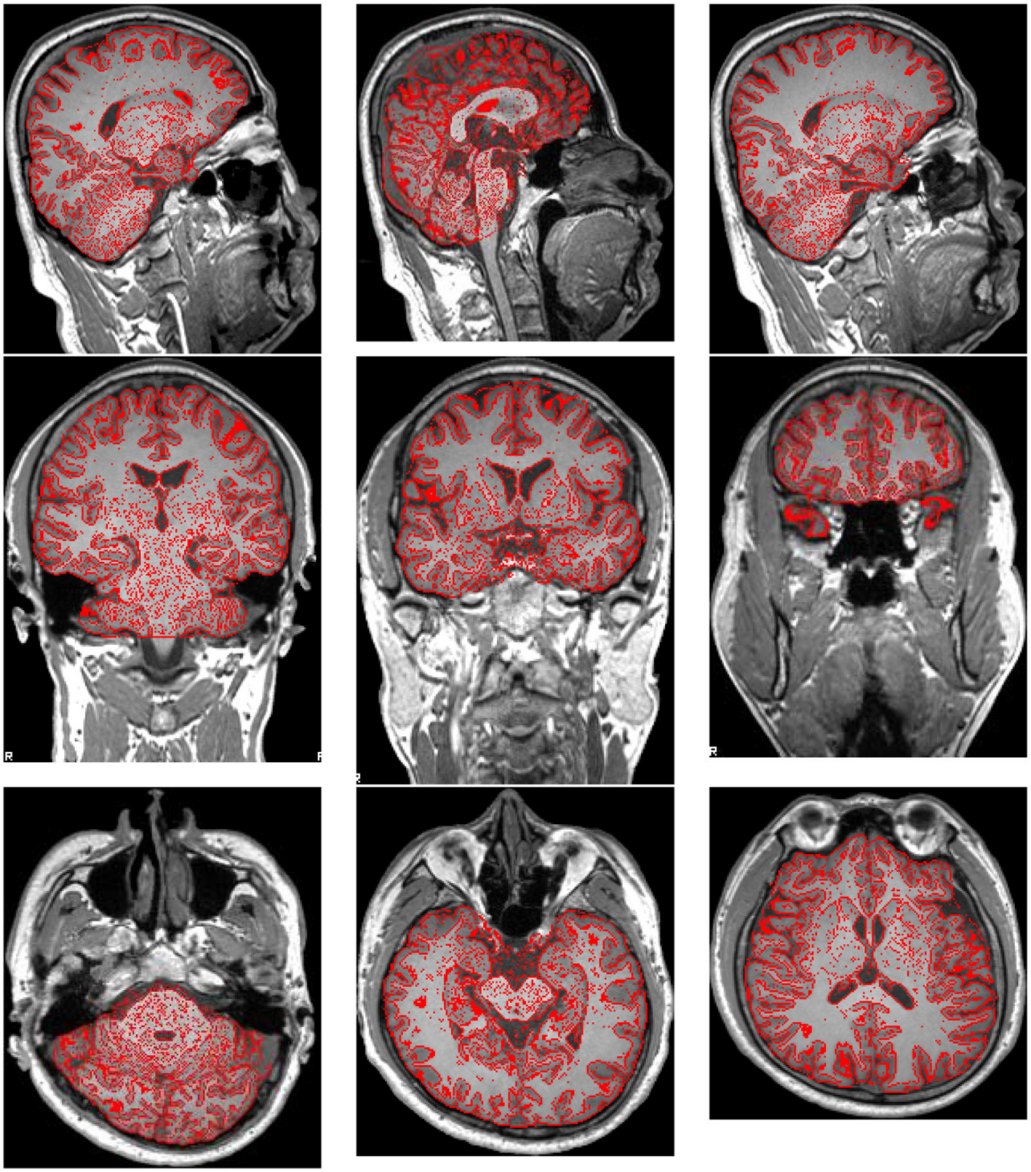
**Brain Extraction Result**.

Preprocessing was performed using FMRIB Software Library (FSL) v5.0. The brain extraction tool (BET) was employed to extract brain and remove skulls. The results were shown in Figure 2, where the red lines outline the edges of extracted brains.

**Figure 3:**
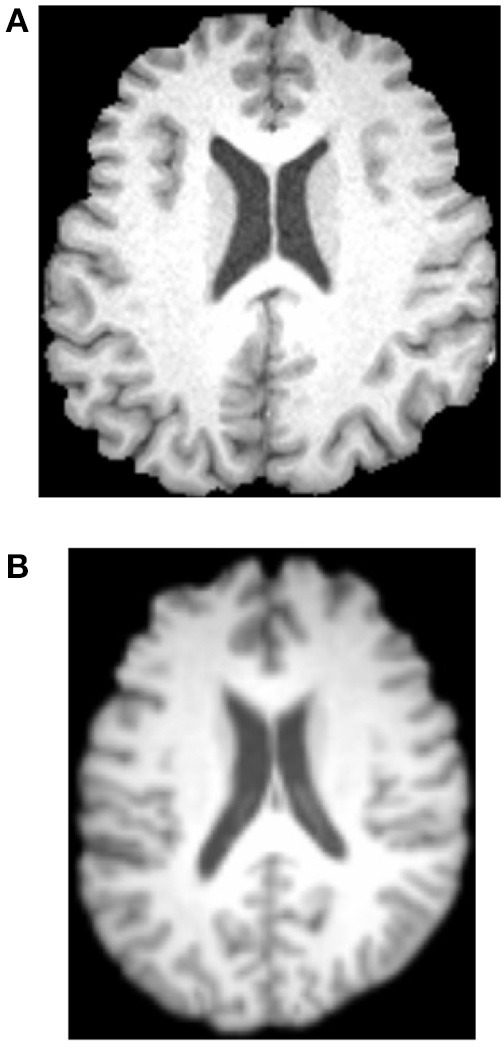
**Normalization and Gaussian kernel results**. **(A)** Before. **(B)** After.

Afterwards, all brains of 49 subjects were normalized to a standard Montreal Neurologic Institute (MNI) template and resampled to 2 mm isotropic voxels using FLIRT and FNIRT tools. Then, the normalized images were smoothed with a Gaussian kernel. Figure 3 shows the results. Three experienced otologists were instructed to select the optimal slice of each patient that covers his/her majority hearing regions, and the selected slice is around 40-th, which contains the significant discrepancy information between SNHLs and HCs. The selected slice of each patient is different.

## Methodology

Computer vision (CV) technique (Wu, 2012a; Ji, 2014; Meireles et al., 2016) is used to help develop the CAD system that can distinguish LHLs and RHLs from HCs. The feature is defined as a piece of information for solving the computational task (here is to detect HL). Traditional CV methods used local features, like edges, corners, blobs, and interesting points & regions (Lee D. H. et al., 2016). Nevertheless, recently researches showed global features may also give the equivalent performances (Li B. et al., 2015). Wavelet entropy (WE) as a novel global feature has attracted attentions from various disciplines.

### Wavelet entropy

WE is a new method developed to analyze transient features of complicated signals (Hosseini et al., 2015; Phillips et al., 2015; Sun, 2015), such as the brain image in this study. The value of WE has a physical meaning of the order/disorder degree of the signal with multiscale time-frequency resolution.

**Figure 4:**
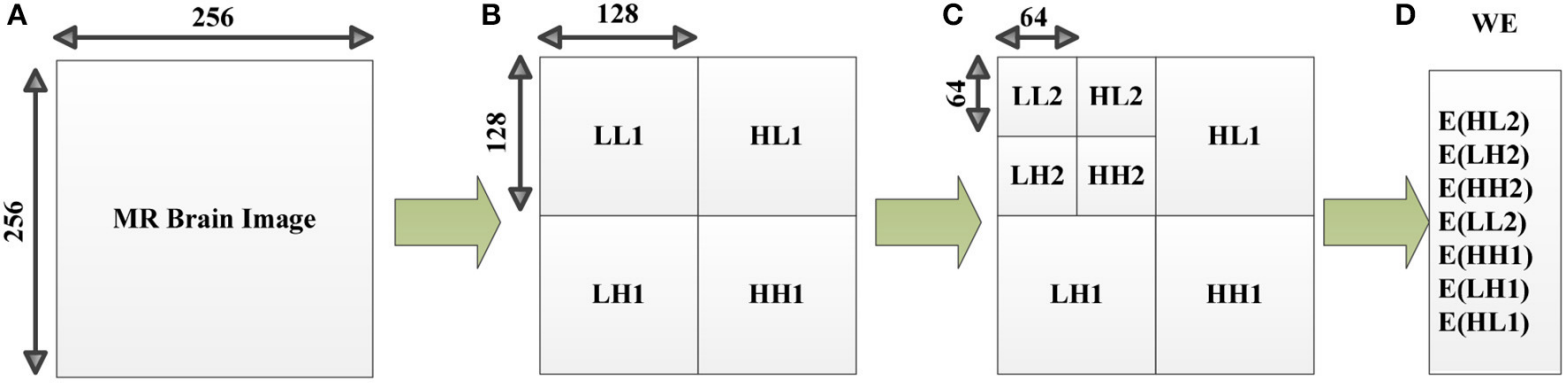
**Diagram of a 2-level decomposition: (A) original MR brain image; (B) one-level decomposition subband; (C) two-level decomposition subband; (D) Wavelet entropy (WE) vector**. **(B,C)** are in wavelet coefficient domain. *L*, low-frequency subband; *H*, high-frequency subband; digits after L/H represents the decomposition level, *E*, entropy; *WE*, wavelet entropy.

WE consists of two steps: discrete wavelet decomposition and entropy calculation (See Figure 4). In the first step, the discrete wavelet decomposition was performed to the given MR brain image, and four subbands (LL1, LH1, HL1, and HH1) were yielded. Here L and H represents the low- and high-frequency coefficients. The LL1 was further decomposed into four smaller subbands as LL2, HL2, LH2, and HH2. Thus, we obtain 3^*^n + 1 subbands for a *n*-level decomposition. In the second step, entropy was calculated over each subband. In total, the WE can reduce a 256 × 256 brain image to a (3^*^n + 1)-element vector. The pseudocode of WE is listed below in Table 2.

**Table 2:** **Pseudocode of wavelet entropy**.

**Algorithm—Wavelet entropy (WE)**
Step 1 Import the brain image
Step 2 Choose the wavelet family and decomposition level *n*
Step 3 Decomposition and generate (3*n* + 1) subbands
Step 4 Calculate entropy over each subband
Step 5 Combine all the entropy results to a column vector and output it as the feature

From Table 2, we know that two factors (wavelet family and decomposition level) are needed to perform a WE. We will discuss the wavelet family in following section and the decomposition level in Section Optimal Decomposition Level.

### Wavelet family

There are many wavelet families: crude wavelets, infinite regular wavelets, orthogonal wavelets, biorthogonal wavelet pairs, and complex wavelets. In this study, we choose a particular case of biorthogonal wavelet pairs (Gawande et al., 2015), viz., B-splines biorthogonal compactly supported wavelet pairs (bior, in short). Compared to other wavelets, bior have excellent advantages of symmetry with FIR filters (Uzinski et al., 2015), vanishing moments for decomposition, and regularity for reconstruction.

**Figure 5:**
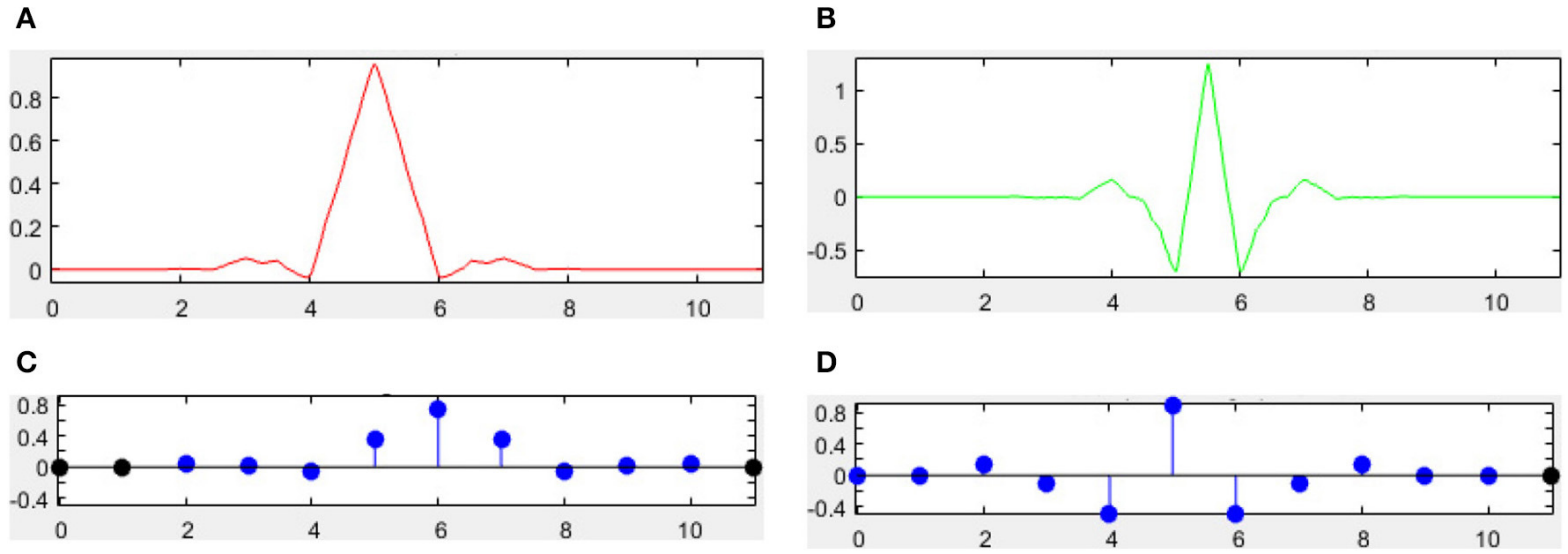
**Decomposition for bior5.5**. **(A)** Scaling Function. **(B)** Wavelet Function. **(C)** Low-pass filter. **(D)** High-pass filter.

**Figure 6:**
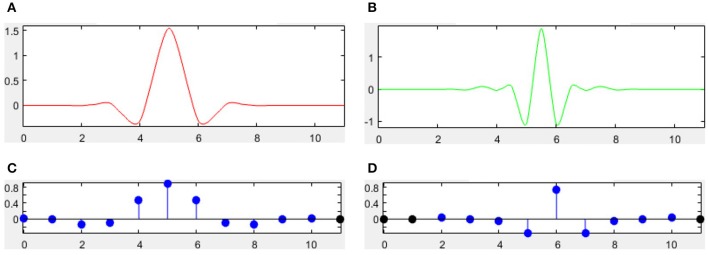
**Reconstruction for bior5.5**. **(A)** Scaling Function. **(B)** Wavelet Function. **(C)** Low-pass filter. **(D)** High-pass filter.

There are many different combinations of parameters for bior wavelets. Usually a “bior r.d” means a B-spline biorthogonal compactly supported wavelet with reconstruction order of *r* and decomposition order of *d*. In this study, we choose the bior5.5 wavelet. Figures 5, 6 shows all relevant functions of decomposition and reconstruction processes for bior5.5.

### Support vector machine

One-level, two-level, three-level, and four-level decompositions of WE were submitted to support vector machine (SVM), to find which level performs the best. SVM is a non-probabilistic binary linear classifier (Yang, 2015b; Berikol et al., 2016; Pal et al., 2016), which belongs to supervised learning model used for regression or classification. Suppose we have an *A*-dimensional *S*-sized dataset as

(1) $$\left\{\left(x_{n},y_{n})|x_{n}\in {\mathbb{R}}^{A},y_{n}\in \{-1,+1\right\}\right\},n=1,...,S$$

where *n* is the index of data. Each *x*_*n*_ is a *A*-dimensional vector and *y*_*n*_ represents its corresponding class label. SVM will build a model by following equation:

(2) $$\underset{w,b}{\min}\frac{1}{2}{\left\|w\right\|}^{2}$$

(3) $$\text{s}.\text{t}.\;y_{n}\left(wx_{n}-b\right)\geq 1$$

where **w** = [*w***]** denotes the weights and **b** = [*b***]** the biases. Note that the distances between two hyperplanes are 2/||**w**||, so formula (2) indicates we need to maximize the distance between two hyperplanes. In the meantime, equation (3) prevents the data falling to the margin as largely as possible.

Soft margin technique (Liu A., 2015) was further introduced for the condition when hyperplane may not split the samples perfectly. The model was then transformed to

(4) $$\begin{array}{l} \underset{w,\xi ,b}{\min}\frac{1}{2}{\left\|w\right\|}^{2}+\varepsilon \sum_{n=1}^{S}\xi_{n}\\ s.t.\;\{\begin{array}{c} y_{n}\left(wx_{n}-b\right)\geq 1-\xi_{n}\\ \xi_{n}\geq 0 \end{array},\;n=1,...,S \end{array}$$

where ξ_*n*_ denotes positive slack variables and ε denotes the error penalty. In the future, some advanced classifiers will be tested, such as nonparallel SVM, fuzzy SVM (Yang, 2015a), kernel SVM (Wu, 2012b), SVM decision tree (Dong, 2014), proximal SVM (Dufrenois and Noyer, 2016), twin SVM (Wang et al., 2016), etc.

### Directed acyclic graph

Remember SVM is only for binary classification, hence, we introduce in the directed acyclic graph (DAG) method to reduce our three-class task (HC, LHL, RHL) to multiple binary classification problems. DAG technique is based on one vs. one approach (Lee J. et al., 2016). Suppose there are in total *C* classes, the DAG constructs individual classifier (IC) for each pair of classes, so in total (*C* − 1)*C*/2 individual classifiers (Gasemyr, 2016).

After each *ij*-th individual classifier is trained with the *i*-th and *j*-th class (*i* = 1,2, …, *C* − 1, *j* = *i* + 1, …, *C*), we submit a new data *x* into each trained individual classifier, obtaining the score (*L*_*ij*_) of *ij*-th individual classifier and its output is the sign function of the score value *L*_*ij*_, viz.,

(5) $$o_{ij}(x)=\text{sgn}(L_{ij}(x))$$

If the score *L*_*ij*_(*x*) is larger than zero, then the output *o*_*ij*_(*x*) is +1, denoting that *x* does not belong to *j*-th class; otherwise output is −1, denoting *x* does not belong to *i*-th class (Joutsijoki et al., 2015).

**Figure 7:**
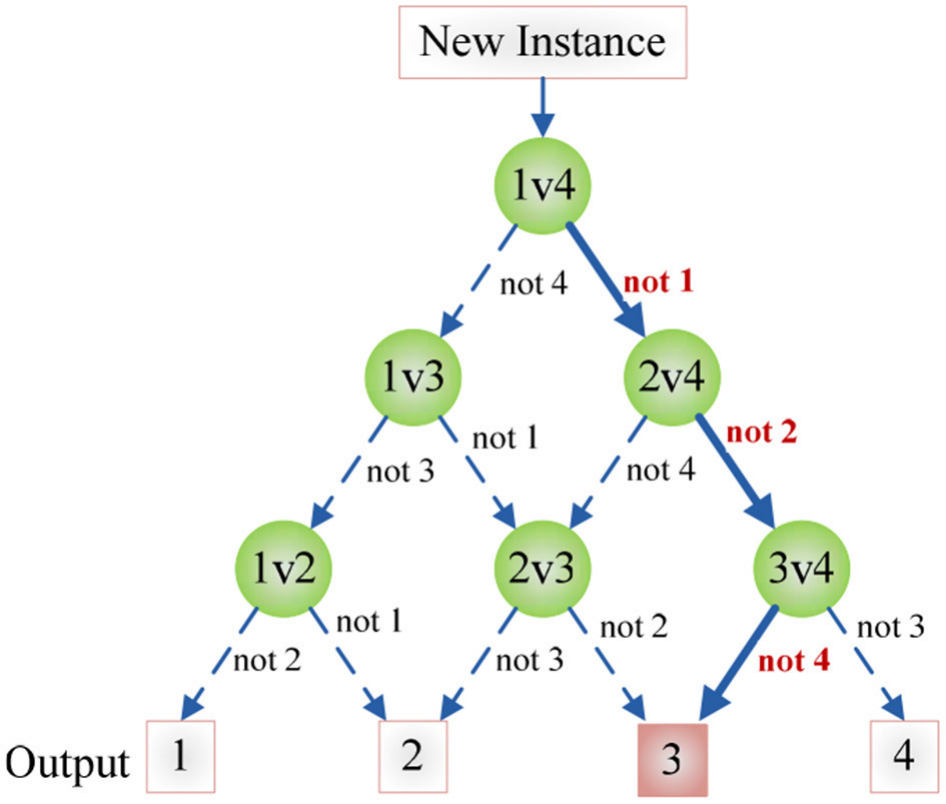
**An example of DAG Technique with *C* = 4**. Root node and intermediate nodes represent individual classifiers, and the leaf nodes represented the output label.

Figure 7 shows an example of using DAG technique to classify from *C* = 4 classes. The “1v4” individual classifier notifies that *x* does not belong to 1-st class, then the “2v4” individual classifier indicates that *x* does not belong to 2-nd class, finally the “3v4” individual classifier tells that *x* does not belong to 4-th class. Obviously, *x* belongs to 3-rd class. Our method is different from Dietl and Weiss (2004). They employed wavelet packets and SVMs to detect cochlear hearing loss. They classified pantonal and high-frequency hearing loss from normal controls. However, they did not use the imaging data.

### Experiments

**Figure 8:**
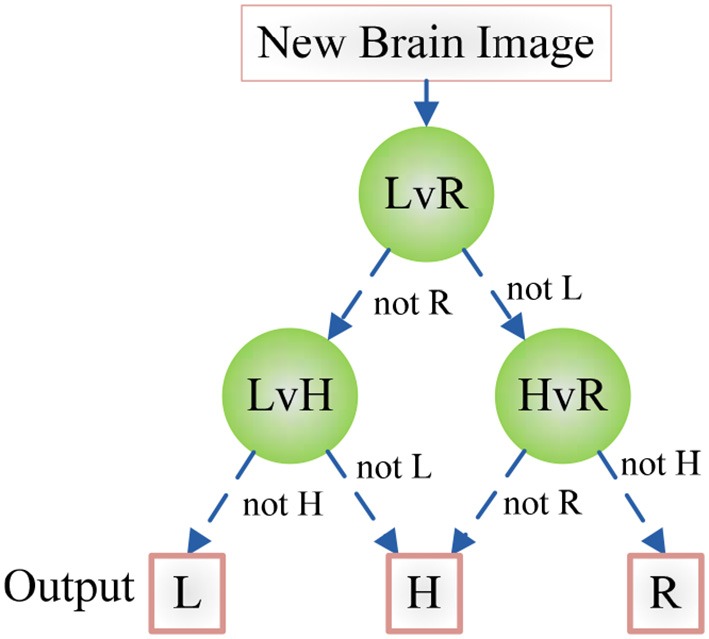
**Diagram of the DAG-SVM for the hearing loss classification**. *L*, LHL; *R*, RHL; *H*, HC.

The CAD system was in-house developed using Matlab 2015a, and run on IBM laptop with 3 GHz Intel i3 dual-processor and 8 GB RAM. With the help of DAG, the classifier is decomposed as three binary classification problems as shown in Figure 8. Here we establish three classifiers, LHL-v-RHL, LHL-v-HC, and HC-v-RHL. The three classifiers are then connected in the style of DAG.

A 10 × 10-fold cross validation were implemented for statistical analysis. First, we divide the dataset into 10-folds, 9-folds for training 1-fold for validation. DAG-SVM was trained each time and the confusion matrixes over the validation fold were combined to form a full confusion matrix. Then, the above procedure repeats 10 times.

The parameters of SVM are set as follows: ε equals to 0.05 by experience, *C* equals to 2 since each individual SVM handles a two-class classification problem. *S* varies in each run since the sum of the sizes of nine training folds are different, but we list the detailed statistical result in **Table 4**.

## Results and discussions

### Wavelet decomposition

**Figure 9:**
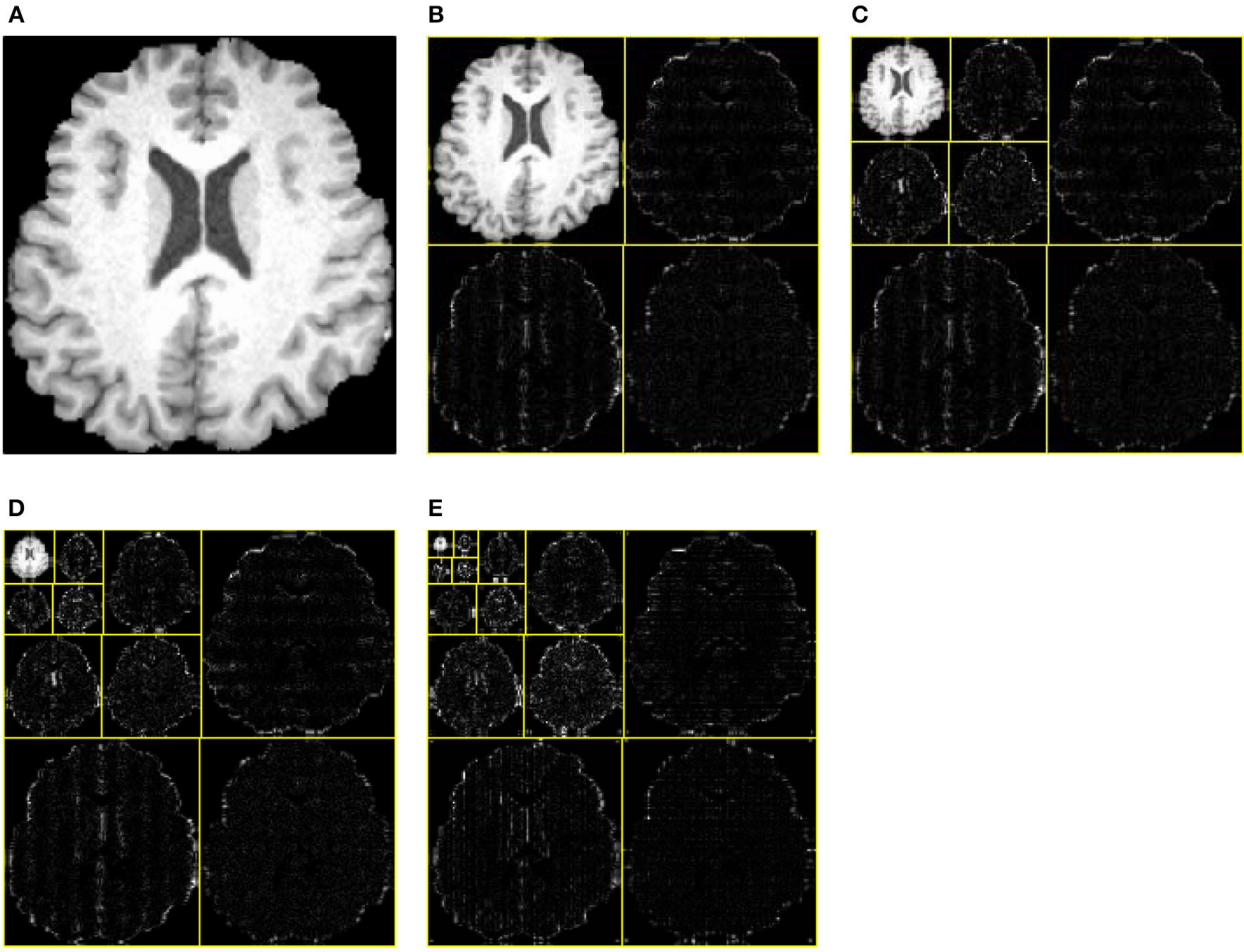
**Decomposition results**. **(A)** Original Image. **(B)** 1-level decomposition. **(C)** 2-level decomposition. **(D)** 3-level decomposition. **(E)** 4-level decomposition.

One, two, and three levels decomposition was implemented over all brain images. Figure 9A shows the original brain image. Figure 9B shows the 1-level decomposition with four subbands. Figure 9C shows the 2-level decomposition with seven subbands. Figures 9D,E show the 3-level with 10 subbands, and 4-level with 13 subbands, respectively.

### Optimal decomposition level

To decide which decomposition level performs the best for our task, we submit the generated 4, 7, 10, and 13 WEs in the last step to the DAG-SVM classifier. The overall accuracy was listed in Table 3 and it was defined as the ratio between the number of correctly classified brains and total brains. Four WEs obtains an accuracy of 92.24%, 7 WEs obtains an accuracy of 94.08%, 10 WEs obtains the highest accuracy of 95.10%, and 13 WEs obtains an accuracy of 94.29%.

**Table 3:** **Classification Accuracy vs. decomposition level**.

**Decomposition level**	**WE Number**	**Overall Accuracy**
1	4	92.24%
2	7	94.08%
3	10	**95.10%**
4	13	94.29%

Bold represents the best.

### Statistical analysis

Table 4 illustrates how we obtain the result of 95.10% for a three-level WE. Each row shows the results of different runs, and each column shows the result of different folds. In the last column, we calculated the accuracy of each run. In the last row, we averaged the results of each run.

**Table 4:** **The experiment results of 3-level decomposition**.

	**F1**	**F2**	**F3**	**F4**	**F5**	**F6**	**F7**	**F8**	**F9**	**F10**	**Total**	**Acc. (%)**
Run 1	3 (4)	5 (5)	5 (5)	4 (5)	5 (5)	5 (5)	5 (5)	5 (5)	5 (5)	5 (5)	47 (49)	95.92
Run 2	3 (4)	5 (5)	5 (5)	5 (5)	5 (5)	5 (5)	5 (5)	5 (5)	5 (5)	5 (5)	48 (49)	97.96
Run 3	4 (4)	5 (5)	5 (5)	5 (5)	5 (5)	5 (5)	5 (5)	5 (5)	5 (5)	4 (5)	48 (49)	97.96
Run 4	4 (4)	5 (5)	5 (5)	5 (5)	5 (5)	5 (5)	5 (5)	3 (5)	5 (5)	5 (5)	47 (49)	95.92
Run 5	4 (4)	5 (5)	5 (5)	5 (5)	5 (5)	4 (5)	5 (5)	5 (5)	5 (5)	5 (5)	48 (49)	97.96
Run 6	2 (4)	5 (5)	5 (5)	5 (5)	5 (5)	5 (5)	5 (5)	4 (5)	5 (5)	5 (5)	46 (49)	93.88
Run 7	4 (4)	5 (5)	5 (5)	5 (5)	5 (5)	5 (5)	5 (5)	5 (5)	4 (5)	5 (5)	48 (49)	97.96
Run 8	4 (4)	3 (5)	5 (5)	5 (5)	5 (5)	4 (5)	5 (5)	5 (5)	5 (5)	5 (5)	46 (49)	93.88
Run 9	4 (4)	5 (5)	5 (5)	5 (5)	4 (5)	5 (5)	5 (5)	5 (5)	4 (5)	4 (5)	46 (49)	93.88
Run 10	4 (4)	4 (5)	5 (5)	4 (5)	4 (5)	5 (5)	4 (5)	4 (5)	5 (5)	3 (5)	42 (49)	85.71
Average												95.10

F, Fold; x(y) means our classifier correctly predicts x brains out of y brains, Acc. means the accuracy for every run, Average gives the averaged accuracy over 10 runs.

### Confusion matrix

The confusion matrix over 10 runs is drawn in Table 5. We read the table in a row-wise way. The second row indicates that 194 HCs are recognized correctly, 4 HCs are recognized as LHLs, and 2 HCs are recognized as RHLs. The third row indicates that 141 LHLs are recognized correctly, 6 LHLs are recognized as HCs, 3 LHLs are recognized as RHLs. The final row indicates that 131 RHLs are recognized correctly, 4 RHLs are recognized as HCs, and 5 RHLs are recognized as LHLs. Table 6 lists the performances over each single class (i.e., a single class versus the combination of other two classes), which can be directly calculated from Table 5.

**Table 5:** **Confusion Matrix**.

	**HC**	**LHL**	**RHL**
HC	194	4	2
LHL	6	141	3
RHL	4	5	131

HC, healthy control; LHL, left-sided hearing loss; RHL, right-sided hearing loss.

**Table 6:** **Performance over each class**.

**Class**	**Sensitivity**	**Specificity**	**Precision**	**Accuracy**
HC	97.00%	96.55%	95.10%	96.73%
LHL	94.00%	97.35%	94.00%	96.33%
RHL	93.57%	98.57%	96.32%	97.14%

HC, healthy control; LHL, left-sided hearing loss; RHL, right-sided hearing loss.

### Comparison with manual method

We compared our method with manual method. Three experienced observers (O1, O2, O3) with clinical experiences longer than 10 years in neuroradiology are invited to give decisions over those 49 subjects, and their reports are listed in Table 7. The accuracies of three observers are of 36.73, 32.65, and 38.78%, respectively.

**Table 7:** **Comparison of accuracy with manual interpretation**.

**O1**	**O2**	**O3**	**Our Method**
36.73%	32.65%	38.78%	95.10%

O, Observer.

### Comparison with state-of-the-art method

There are many other popular classifiers used for classifying MR images. Kale et al. (2013) employed feedforward neural network (FNN). Scherfler et al. (2016) used decision tree (DT) as the classifier. Vasta et al. (2016) utilized naive Bayesian classifier (NBC). In this study, we compared the proposed DAG-SVM with FNN (Kale et al., 2013), DT (Scherfler et al., 2016), and NBC (Vasta et al., 2016). The features were the same as three-level WEs, the statistical analysis is all set to 10 × 10-fold cross validation, and the optimal parameters of classifiers were obtained by grid searching. The results are listed in Table 8.

**Table 8:** **Classifier comparison**.

**Classifier**	**Accuracy**
FNN (Kale et al., 2013)	94.08%
DT (Scherfler et al., 2016)	91.84%
NBC (Vasta et al., 2016)	91.02%
DAG-SVM (Proposed)	95.10%

## Discussions

The WEs in Figure 9 were obtained after performing entropy calculation over each subband, namely, 4 WEs for 1-level decomposition, 7 WEs for 2-level decomposition, 10 WEs for 3-level decomposition, and 13 WEs for 4-level decomposition. As it expects, more WEs will provide more information. Nevertheless, too many WEs will deteriorate the performance of the classifier. In this paper, we tested 4, 7, 10, and 13 WEs.

The support vectors are difficult to display due to four reasons: (i) They are in high-dimensional feature space; (ii) The classifier is regarded as a “black box” from the view of computer scientists; (iii) We have three individual classifiers, and their support vectors are different; (iv) The 10 × 10-fold statistical analysis make us to run the classifier training 100 times, and the support vectors at each time are different. Further, several other research teams also used SVMs without displaying the support vectors (Chen et al., 2015; Liu G., 2015; Tan et al., 2015; Chen M. Y. et al., 2016). This is like a face recognition system that recognizes faces quite well with a too complicated inner structure to display.

A limitation is that the wavelet subbands are mathematically generated, thus the results only have mathematical meaning but they cannot implicate which brain area drives the difference between the classes. Traditionally, scholars like to measure the cortical thickness (Marie et al., 2016), generate gray or white matter maps (Bonnier et al., 2016), since it carries more information and links better between classification performance and brain regions. But nowadays, the growth of artificial intelligence has a trend of creating “black box” model that let the machine extract a set of task-oriented image features automatically (Premaladha and Ravichandran, 2016), with better classification performance than traditional methods. Therefore, our method follows the new idea of classification.

Table 3 shows the overall accuracy results for each decomposition level. The results indicate 3-level decomposition with 10 WEs performed the best with the highest accuracy of 95.10%. Again, this falls in line with our expectation. First, adding WEs will provide more information, which do good to the classifier. However, too many features may confuse the classifier. From Figure 9E, we can see that the smallest subbands of 4-level decomposition generates is unclear and may be blurred by neighboring subbands.

Table 6 shows that HC has a sensitivity of 97.00%, a specificity of 96.55, a precision of 95.10%, and an accuracy of 96.73%. LHL has a sensitivity of 94.00%, a specificity of 97.35%, a precision of 94.00%, and an accuracy of 96.33%. RHL has a sensitivity of 93.57%, a specificity of 98.57%, a precision of 96.32%, and an accuracy of 97.14%. We found that computer can detect all three classes with an accuracy higher than 96%. This indicates our method achieved excellent result. The high accuracy stems from two facts: (i) LHL patients are different from RHL patients (Fan et al., 2015); and (ii) SNHLs have difference with healthy subjects in brain structures (Yang et al., 2014). The situation was similar to the finger tipping task in fMRI, which the activation of left hand and that of right hand is distinctive for computers (Kuehn et al., 2015; Sun et al., 2015).

Table 7 shows that computer programs may replace human interpretation in terms of brain MR images. The reason why those three experienced observers fail lies in the difference between SNHL and HC are difficult to be perceived by human eye, through which the brain MR images of HL patients appear normal. The accuracies obtained by human observers are close to baseline demonstrated this point. This also validated the success of our CAD system, which is due to the high-sensitivity of computers for slight pixel gray-level difference and region atrophy.

Table 8 shows FNN (Kale et al., 2013) achieves an accuracy of 94.08%, DT (Scherfler et al., 2016) achieves an accuracy of 91.84%, and NBC (Vasta et al., 2016) achieves an accuracy of 91.02%. We can see our method “DAG-SVM” obtains the highest accuracy of 95.10%. The result shows the superiority of SVM to other popular classifiers.

## Conclusions

In this paper, we developed a novel CAD for detecting unilateral hearing loss. To the best known of the authors, we are the first to apply SVM in UHL detection. The overall accuracy of the three-class problem is 95.10%, which offers a promising result.

In the future, we will consider other classification tasks, such as including the classes of pan-tonal and high-frequency hearing loss. Another research direction is to use advanced optimization techniques to train SVM, such as hybrid genetic algorithm (Lu, 2016), biogeography-based optimization (Wei, 2015), particle swarm optimization (PSO, Ji, 2015), chaotic adaptive PSO (Wu J., 2016), etc.

More feature selection methods will be tested such as displacement field (Wang et al., 2015), eigenbrain (Phillips, 2016), and fractional Fourier entropy (Sun, 2016). More classifiers will be tested, for example, the k-nearest neighbors (Zhou, 2016), artificial neural network (Wu X., 2016), decision tree (Zhang, 2014), etc.

## Author contributions

SW, MY, and YZ conceived the study. SD, JY, and YZ designed model. MY and BL acquired the data. SW, JG, and YZ analyzed the data. JR and YZ processed the data. MY, SD, TY, and YZ interpreted the results. SW and YZ developed programs. SW, TY, and YZ wrote the draft. All authors gave critical revisions.

### Conflict of interest statement

The authors declare that the research was conducted in the absence of any commercial or financial relationships that could be construed as a potential conflict of interest.

## Abbreviations

SNHL: Sensorineural hearing loss
UHL: Unilateral hearing loss
LHL: Left-sided hearing loss
RHL: Right-sided hearing loss
HC: Healthy control
MRI: Magnetic resonance imaging
PTA: Pure tone average
MNI: Montreal neurologic institute
DAG: Directed acyclic graph
FNN: Feedforward neural network
DT: Decision tree
NBC: Naive Bayesian classifier.
